# An improved semantic segmentation algorithm for high-resolution remote sensing images based on DeepLabv3+

**DOI:** 10.1038/s41598-024-60375-1

**Published:** 2024-04-27

**Authors:** Yan Wang, Ling Yang, Xinzhan Liu, Pengfei Yan

**Affiliations:** 1 College of Geography and Environmental Science, Henan University, Kaifeng, China; 2 Key Laboratory of Geospatial Technology for Middle and Lower Yellow River Regions, Ministry of Education, Kaifeng, China

**Keywords:** Information technology, Environmental sciences

## Abstract

High-precision and high-efficiency Semantic segmentation of high-resolution remote sensing images is a challenge. Existing models typically require a significant amount of training data to achieve good classification results and have numerous training parameters. A novel model called MST-DeepLabv3+ was suggested in this paper for remote sensing image classification. It’s based on the DeepLabv3+ and can produce better results with fewer train parameters. MST-DeepLabv3+ made three improvements: (1) Reducing the number of model parameters by substituting MobileNetV2 for the Xception in the DeepLabv3+’s backbone network. (2) Adding the attention mechanism module SENet to increase the precision of semantic segmentation. (3) Increasing Transfer Learning to enhance the model's capacity to recognize features, and raise the segmentation accuracy. MST-DeepLabv3+ was tested on international society for photogrammetry and remote sensing (ISPRS) dataset, Gaofen image dataset (GID), and practically applied to the Taikang cultivated land dataset. On the ISPRS dataset, the mean intersection over union (MIoU), overall accuracy (OA), Precision, Recall, and F1-score are 82.47%, 92.13%, 90.34%, 90.12%, and 90.23%, respectively. On the GID dataset, these values are 73.44%, 85.58%, 84.10%, 84.86%, and 84.48%, respectively. The results were as high as 90.77%, 95.47%, 95.28%, 95.02%, and 95.15% on the Taikang cultivated land dataset. The experimental results indicate that MST-DeepLabv3+ effectively improves the accuracy of semantic segmentation of remote sensing images, recognizes the edge information with more completeness, and significantly reduces the parameter size.

## Introduction

Remote sensing technology has gradually replaced conventional manual regional survey methods due to its wide monitoring range, quick data acquisition, and a large amount of obtained information. It is extensively used in soil research^1^, geological engineering^2^, land resources^3^, and other fields. The quality of remote sensing images has increased along with the rapid development of the technology. Remote sensing images can provide a wealth of information about ground objects, such as ground vegetation cover, ground temperature, and land use. Semantic segmentation of remote sensing images is the fundamental and critical component of understanding and analyzing remote sensing images, which converts complex remote sensing images into feature classification information that can be understood and processed to support practical applications. As a result, the semantic segmentation technique for remote sensing images has significant research implications.

The development of machine learning algorithms brings significant changes to remote sensing image classification. Traditional machine learning approaches include Decision Tree^4^, Support Vector Machine (SVM)^5^, Random Forest (RF)^6^, Conditional Random Field (CRF)^7^, and others. Li et al.^8^ combined color features with a support vector machine classifier to detect multiple classes of features in remote sensing images. Volpi et al.^9^ used a structured support vector machine to classify urban scenes. Sun et al.^10^ employed random forest integrated learning techniques to categorize the pixels of remote sensing images, then enhanced the classification findings with an improved conditional random field. Most traditional machine learning-based remote sensing image interpretation algorithms adopt feature extraction and feature analysis, and the interpretation effect is good for specific scenes and datasets^11^. However, classic machine learning algorithms have restricted feature extraction and cannot accurately capture the nuances of the input^12^. When the background level of the remote sensing image to be processed is complicated and the target scale has large fluctuations, the model accuracy suffers and under-fitting or over-fitting occurs.

High-resolution remote sensing images can give rich feature information and finely present the spatial structure and textural features due to their complex and diversified information, rich features, and vast size^13^. However, while high-resolution remote sensing images provide more data and information, they also pose significant challenges to remote sensing image interpretation, such as high interpretation costs, time-consuming, which makes it difficult to meet the urgent demand for rapid extraction and updating of resource information at present. Because of the rapid advancement of artificial intelligence technology, semantic segmentation algorithms are widely used in natural image processing^14–16^. The method based on the convolutional neural network (CNN) was gradually adopted in remote sensing image interpretation^17^. Zhu et al.^18^ compared the GoogLeNet model to the SVM method for the extraction of urban construction land in Landsat8 remote sensing images to demonstrate the advantages of deep learning for construction land. Jadhav et al.^19^ used a ResNet101 network for automatic semantic segmentation of high-resolution remote sensing images for land cover and crop type to achieve classification accuracy of major crops. Kussul et al.^20^ demonstrated that the design with an ensemble of convolutional neural networks (CNNs) performs better than the one with multilayer perceptrons(MLPs) in distinguishing crop types in remote sensing images.

In comparison to conventional machine learning techniques, CNNs have significantly increased the segmentation accuracy of remote sensing images, but the classical CNN model has redundant computations during the batch operation, which will result in higher memory consumption and lower segmentation efficiency^21^. Therefore, researchers have created a variety of improvements based on convolutional neural networks. Fully Convolutional Networks (FCN) were suggested by Long^22^ and replaced CNN's fully connected layers with convolutional layers to produce images with contextual spatial features. Fu et al.^23^ optimized the FCN model by using the atrous convolution and used the conditional random field to post-process the segmented data, which greatly improved the segmentation accuracy. To increase algorithm accuracy and reduce the impact of noise, Chen et al.^24^ used the method of overlapping the SNFCN and SDFCN semantic segmentation frameworks based on the shortcut-block structure, which significantly increased remote sensing accuracy in urban areas.

However, FCN does not consider the relationship between pixels while upsampling, which could result in information loss, and the segmentation results are still rough. To improve segmentation accuracy, numerous improved models based on the FCN were invented one after the other. For example, the UNet network with a U-shaped structure proposed by Ronneberger^25^ uses an encoder to generate deep semantic information, a decoder to recover image spatial resolution, and a jump connection to splice and fuse deep abstract features with shallow detailed features in each level to integrate more feature information than the FCN, resulting in more accurate pixel boundary localization and significantly improved segmentation accuracy. The SegNet network proposed by Badrinarayanan^26^ is also an encoder-decoder structure. Unlike the FCN network, which directly copies the feature maps, the decoder upsamples the low-resolution feature maps by pooling indexes with fewer training parameters, which has great advantages in storage and computational efficiency. Weng et al.^27^ applied the separable residual module to SegNet for water body segmentation, and the accuracy was greatly increased compared with FCN. Zhao et al.^28^ suggested the PSPNet network with a pyramidal pooling structure, which separates the feature map into multiple levels and sub-regions, combines the context data from various regions, completes multi-level semantic feature fusion, and mines global data completely.

DeepLab networks^29–32^ are deep learning networks open-sourced by the Google research team, which has introduced Atrous Convolution^33^, Conditional Random Field (CRF)^7^, and Atrous Spatial Pyramid Pooling(ASPP)^34^ modules in succession. These modules fully utilize the feature graph's multi-scale information, enhancing the model's ability to capture fine details and raising the performance of the deep learning semantic segmentation network to a new level. DeepLabv3+ adds a simple but effective decoder module based on DeepLabv3, which improves the model’s effect in dealing with image boundaries and better preserves the target's edge details. The resolution of coding features can be output using the proposed encoder-decoder structure by controlling the atrous convolution, and the accuracy and running time can be balanced.

DeepLabv3+ is one of the best general segmentation networks available today, with a smooth segmentation edge and segmentation accuracy that leads in a number of public datasets^31^. However, there are still some problems. First, the DeepLabv3+ encoder’s model Xception^35^ has a complex structure, requiring a large amount of parameter calculation and memory, resulting in slow fitting speed and low segmentation efficiency; second, it is hard to precisely capture the contour of ground objects in semantic segmentation of high-resolution remote sensing images, small targets are missed, and similar objects are easily misjudged, resulting in low segmentation accuracy.

In light of the aforementioned issues, the three improvements made to the DeepLabv3+ network in this paper are as follows:

1. At the coding layer, the DeepLabv3+ model’s feature extraction module Xception network is replaced with a lightweight network MobileNetV2^36^ to reduce the number of parameters in the semantic segmentation model and improve model training efficiency.
2. The SENet^37^ is added to distribute channel weight and improve the problem of missed segmentation and target misjudgment, thereby increasing segmentation accuracy.
3. Transfer learning^38^ is added to the original model, and the model obtained from the ImageNet^39^ dataset is used as the pre-training model to improve the model's capacity to collect features and promote network segmentation accuracy.

The remainder of this paper is structured as follows: the datasets and pre-processing methods are described in section “Data”. The DeepLabv3+ model, the MST-DeepLabv3+ model, and the semantic segmentation evaluation metrics are covered in section “Methods”. In section “Experimental results and analysis”, the experimental configuration is then briefly introduced, and the results are thoroughly analyzed. The findings of the experiment are discussed in section “Discussion”. This paper is concluded in section “Conclusions”, which also outlines some potential research topics.

## Data

### ISPRS dataset

The ISPRS dataset^40^ contains two sub-datasets, Vaihingen and Postdam, both of which cover the majority of the urban scenes. The Vaihingen dataset includes 33 different sizes of remote sensing images extracted from a larger top-level orthoimage. The top-level image and DSM (Digital Surface Model) have a spatial resolution of 9 cm. The remote sensing images consist of three bands: near-infrared, red, and green. The Postdam dataset has 38 UAV (Unmanned Aerial Vehicle) images with a resolution of 5 cm, which are 6000 pixels × 6000 pixels. Both datasets were manually classified into the six land cover types: background, impervious surface, tree, building, car, and low vegetation.

This paper makes use of the entire Vaihingen dataset. Since the dataset is small, this paper expands it by flipping, cropping, and rotating, finally obtains 3720 images, randomly selects 2976 images of which are used as the training set and 744 images are used as the test set. All of the images are 512 pixels × 512 pixels in size.

### GID dataset

The GID dataset^41^ is a large-scale high-resolution remote sensing image land cover data set based on data collected by the Chinese Gaofen-2 satellite. The GID dataset includes two parts: the large-scale classification dataset and the land-cover dataset, both of which have a large number of samples from the same region, different seasons, and different light conditions, and are very close to the true distribution characteristics of ground features. 150 images from over 60 different Chinese cities are included in the large-scale classification dataset, which spans an area of over 50,000 square kilometers. The size of each image is 6800 pixels × 7200 pixels, with a spatial resolution of 1 m. The land cover categories in the large-scale classification dataset are water, built-up, farmland, meadow and forest. The land-cover dataset includes 15 categories. There are 30,000 image blocks in total.

The large-scale classification dataset of the GID is used for the experiments in the paper. 150 images are cropped to 512 pixels × 512 pixels without overlap, and 27,300 images are obtained, 80% of which are randomly used as the training set and 20% as the test set.

### Taikang cultivated land dataset

To verify the feasibility of the MST-DeepLabv3+ model in practice, we selected high-resolution images from the Gaofen-1 remote sensing satellite in Taikang County, Zhoukou City, Henan Province, China, and created a dataset named Taikang cultivated land dataset for land use classification. The Gaofen-1 remote sensing satellite images of the study area are from the Gaofen Hubei Center^42^.

The Gaofen-1 satellite is an Earth observation remote sensing satellite independently developed by China. It has an average orbital altitude of 644.5 km and a lifespan of 5–8 years. It is equipped with two 2 m resolution panchromatic and 8 m resolution multispectral PMS cameras and four 16m resolution WFV camera, coverage period is 41 days and 4 days respectively. The ground width of the PMS camera is greater than 60 km, and it has five bands, namely panchromatic (wavelength 0.45–0.90 μm), blue (Band1, 0.45–0.52 μm), green (Band2, 0.52–0.59 μm), and red (Band3, 0.63–0.69 μm) and near-infrared (Band4, 0.77–0.89 μm). The WFV camera has a ground width greater than 800 km and has four bands, namely near-infrared, red, green and blue band^43^.

**Figure 1:**
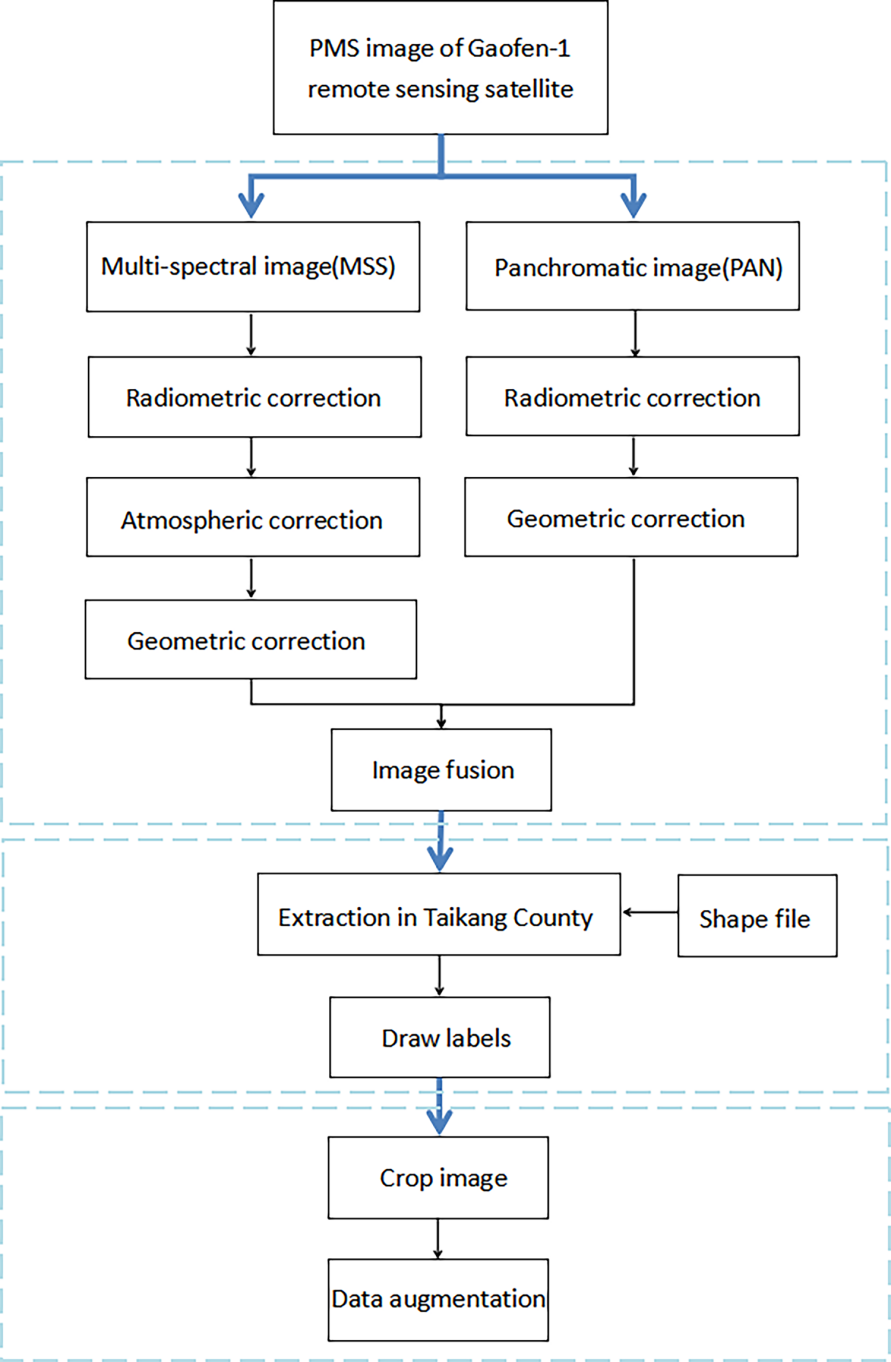
Taikang cultivated land dataset production flow chart.

We selected PMS images with higher resolution for experiments, and filtered the images according to the criteria of clearly visible farmland texture and less cloud coverage. Based on the growth and maturity cycles of farmland crops in the study area, two images in February and May 2017 were finally selected as source data, with scene IDs of 3350241 and 3661472 respectively. The sensors for panchromatic and multispectral images are PAN1 and MSS1 respectively, and the unified projection coordinate system is WGS_1984_UTM_Zone_50N. The dataset creation process is shown in Fig. 1, using ENVI, ArcGIS, and Python tools in turn for preprocessing, drawing labels and cropping. After image processing, 6084 images of 512 pixels × 512 pixels are finally obtained and randomly divided into 5475 training images and 609 prediction images.

## Methods

### DeepLabv3+

**Figure 2:**
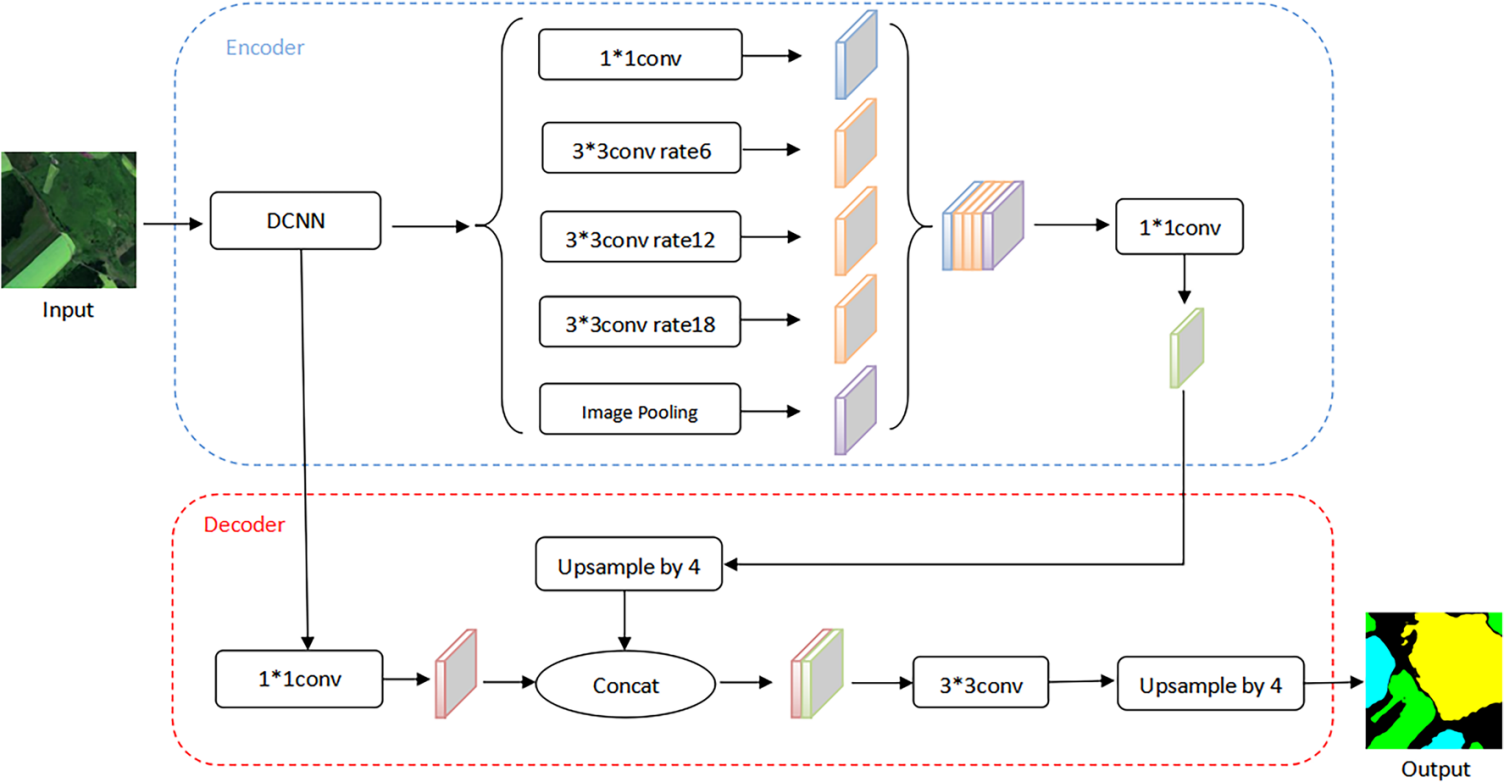
DeepLabv3+ model structure.

DeepLabv3+ model^31^ uses an encoder-decoder structure, with DeepLabv3 serving as the network's encoder, optimizing the extraction effect of the target's edge information, and then using the decoder to recover the feature information and output the predicted results, which improves the segmentation effect and retains the target's edge details. DeepLabv3+ takes the Xception model as the backbone network and applies the deep separable convolution to the ASPP module and the decoder module to create an encoder-decoder network with better segmentation effects. The DeepLabv3+ model’s structure is depicted in Fig. 2.

1. Encoder: Serial atrous convolution is used in the backbone DCNN. After the image passes through the backbone network, the results are provided to the Decoder and ASPP modules for feature extraction, respectively. Serial atrous convolution is used in the backbone DCNN. After the image passes through the backbone network, the results are provided to the Decoder and ASPP modules for feature extraction, respectively. The deep features are extracted by the ASPP module and then merged. It enters the decoder after 1 × 1 convolution is used to change the number of channels;
2. Decoder: After four-fold upsampling of the output of the deep features from the Encoder part, the features are fused with the shallow features that are downsampled using 1 × 1 convolution. And then, the features are further fused using 3 × 3 convolution. Finally, four-fold upsampling is performed using a bilinear interpolation method to get results of the same size as the original image.

### MST-DeepLabv3+

**Figure 3:**
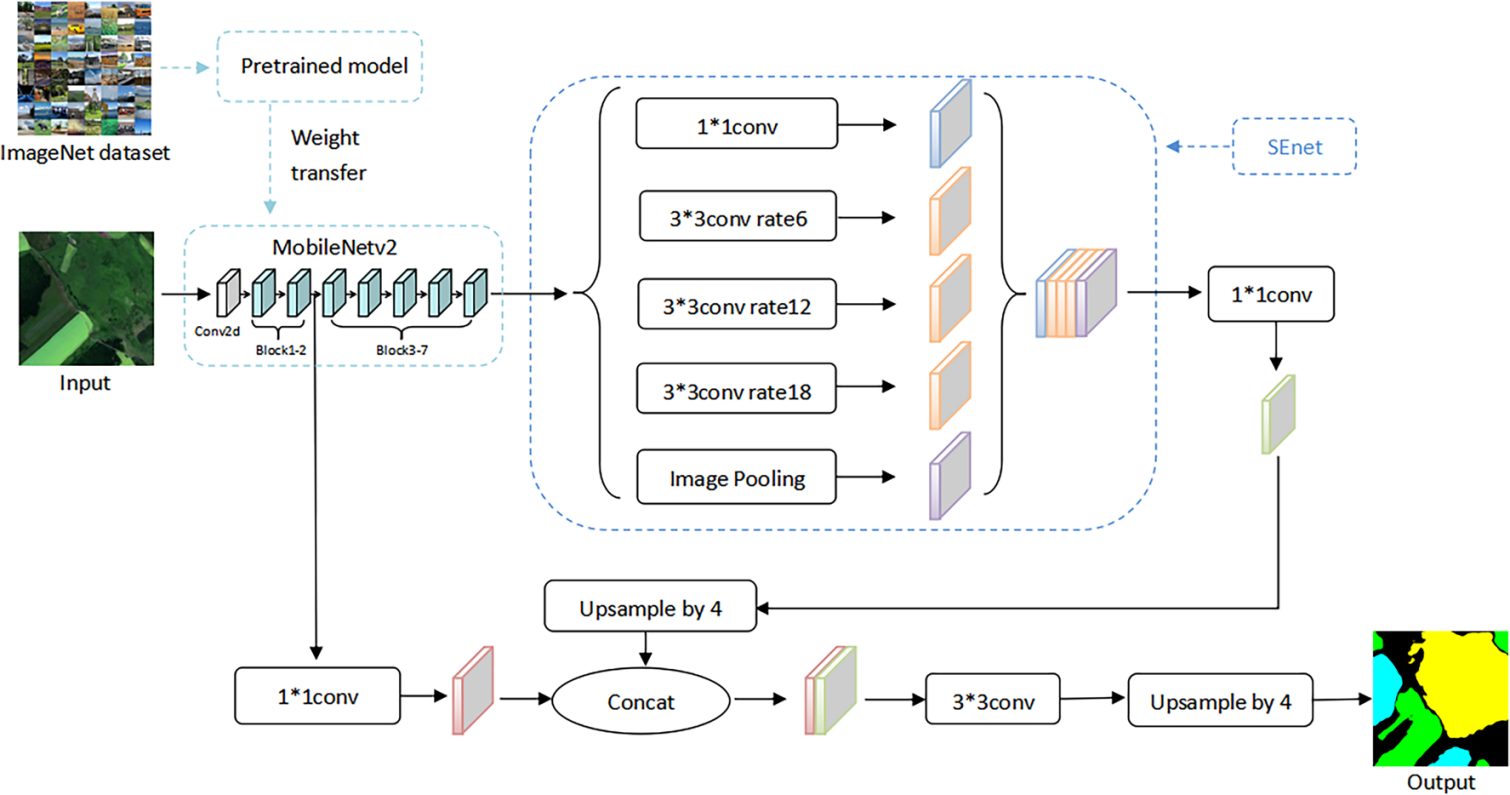
Structure of the MST-DeepLabv3+ model.

This paper suggests a model called MST-DeepLabv3+ that is based on DeepLabv3+. We use the MobileNetV2 network instead of Xception as the backbone network; employ the transfer learning method to reduce the model complexity while improving the segmentation performance; and fuse the attention mechanism at appropriate locations in the network to improve the weight of the feature channel with good network performance, so as to improve the efficiency of remote sensing image semantic segmentation. MST is obtained as an acronym combination of MobileNetV2, SENet and Transfer learning. Figure 3 shows the structure of the MST-DeepLabv3+ model.

#### MobileNetV2

**Figure 4:**
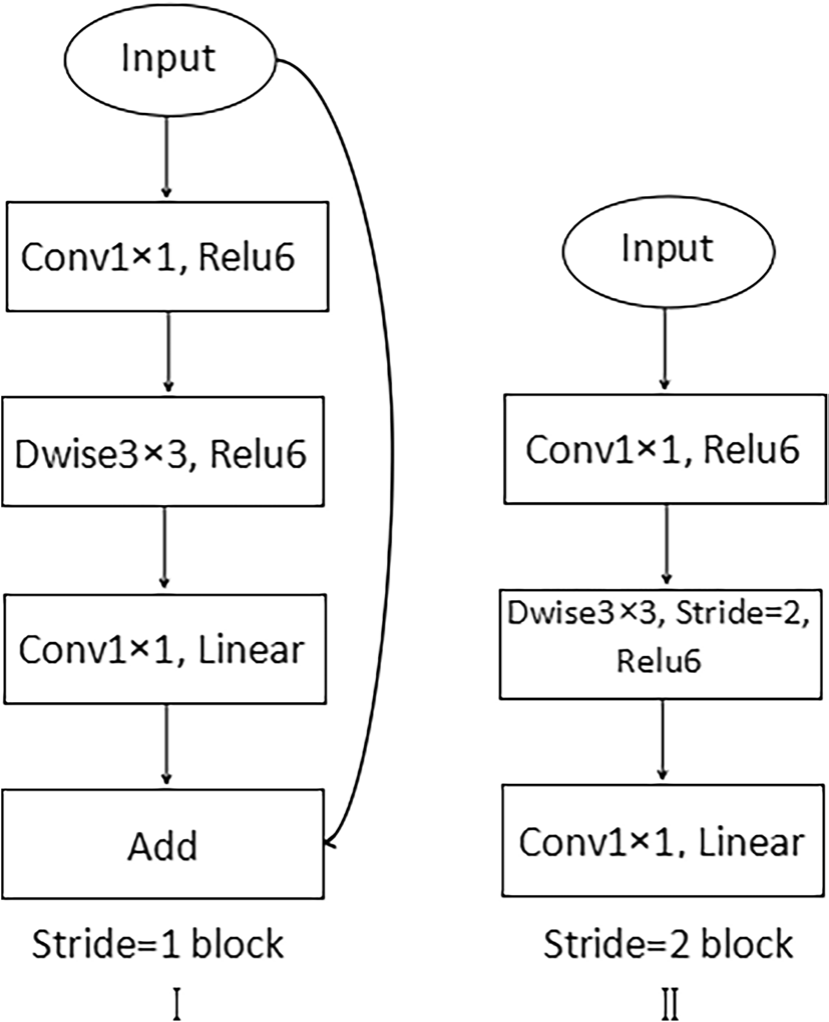
The structure of Inverted residual block.

MobileNetV2 network^36^ uses expansion coefficients to help control the network size. The network structure is deep but less computationally intensive, which can save training resources and has great advantages for target extraction in remote sensing images^44^. MobileNetV2 introduces the structure of inverted residual, as seen in Fig. 4, which increases the dimensionality of the convolution, enhances the model feature extraction ability, and lowers the number of model parameters. Additionally, MobileNetV2 uses the linear bottle-necks structure to prevent information extraction loss due to the destruction of target features by ReLU after dimensionality reduction^36^.

Instead of Xception, we employ the lightweight MobileNetV2 network, which is capable of faster semantic segmentation of remotely sensed images. And the MobileNetV2 network is optimized by deleting the standard convolution operation and the global average pooling layer set by the last three layers to achieve classification, making it more compatible with the DeepLabv3+ model for semantic segmentation operation. And the step size s of the seventh layer is changed from 2 to 1, and only four downsampling operations are performed to ensure the image resolution and segmentation effect. The information of MobileNetV2 network structure used in this paper is shown in Table 1.

**Table 1:** MobileNetV2 network structure.

Input	Operator	t	c	n	s
5122 × 3	conv2d	–	32	1	2
2562 × 32	Bottleneck	1	16	1	1
2562 × 16	Bottleneck	6	24	2	2
1282 × 24	Bottleneck	6	32	3	2
642 × 32	Bottleneck	6	64	4	2
322 × 64	Bottleneck	6	96	3	1
322 × 96	Bottleneck	6	160	3	1
322 × 160	Bottleneck	6	320	1	1

The t stands for the channels’ expansion multiple, the c stands for how many output channels there are, The n indicates how many times the current operator will be repeated, The s is the stride^45^.

#### SENet

**Figure 5:**
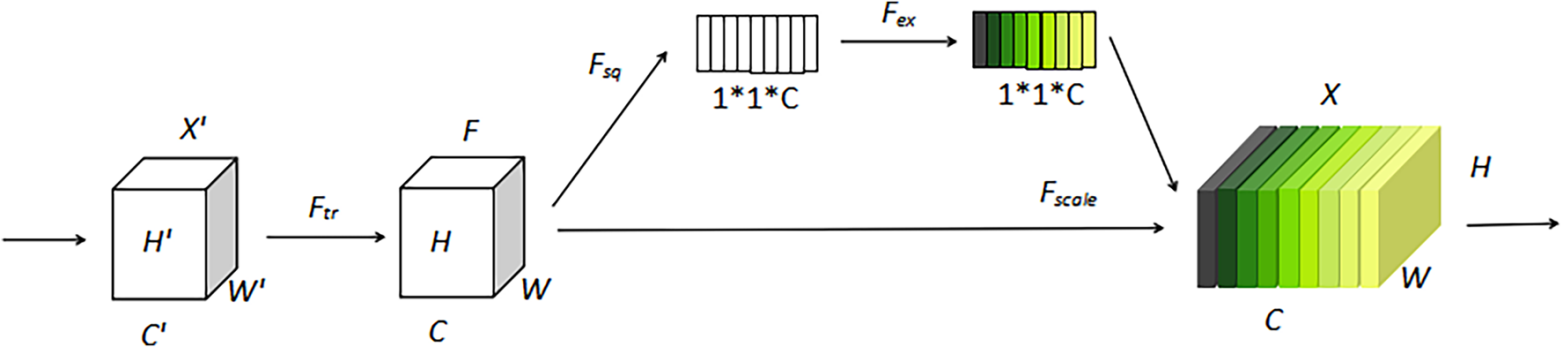
The structure of the SENet.

SENet (Squeeze-and-Excitation Networks)^37^ enables the network to obtain the importance of different feature channels in the feature map and assign weight values to the feature channels according to their importance, so as to focus on certain feature channels. SENet begins with global information to accomplish the goals of emphasizing key traits while suppressing others, as well as to realize the automatic selection and weight distribution of attention regions. In this paper, we add SENet before 1 × 1 convolution in DeepLabv3+’s encoder to reduce the influence of irrelevant features after stitching on recognition accuracy. Different weights are applied to the outputs within the coding region to achieve optimization of the feature map, which brings significant performance improvement to the existing segmentation model with a small additional computational cost. Figure 5 depicts the structure of the SENet.

Squeeze and Excitation are the two operations that makeup SENet. The Squeeze operation is responsible for the global pooling of spatial dimensions, while the Excitation operation learns the pooled channel dependencies and assigns channel weights. The final output of the SENet module is produced by multiplying the output of the Excitation operation by the original input features.

The equation for Squeeze is:

1 $$ z = F_{sq} \left( f \right) = \frac{1}{H \times W}\sum\nolimits_{i = 1}^{H} {\sum\nolimits_{j = 1}^{W} {f\left( {i,j} \right)} } , $$

In the above equation,$f \in R^{H \times W}$ is a two-dimensional feature map set and, *f(i,j)* is one of the elements,* H* and *W* denote the height and width of the feature map spatial information, respectively; z is the Squeeze operation output.

The equation for Excitation is:

2 $$ s = F_{ex} \left( {z,w} \right) = \sigma \left[ {W_{2} \delta \left( {W_{1} z} \right)} \right], $$

In the above equation, *σ* and *δ* denote the Sigmoid and ReLU activation functions, respectively; $W_{1} \in R^{{\frac{c}{r} \times C}}$, $W_{2} \in R^{{C \times \frac{c}{r}}}$, $W_{1}$ and $W_{2}$ are some elements of them, respectively, and *r* is the imensionality reduction coefficient;* s* is the output of Excitation operation.

After the Excitation operation, the resulting output weights are multiplied by the original input features:

3 $$ x = F_{scale} \left( {f,s} \right) = s \cdot f\left( {i,j} \right), $$

In the equation, *x* is a value in the final output X of the SENet. X = [x_1_, x_2_,…, x_c_].

#### Transfer learning

Transfer learning^38^ is a method in deep learning that starts model training on a new dataset with a model that has already been trained on an existing dataset. Usually, when we conduct deep learning experiments, the model’s parameters, such as its weights and biases, are generated by the system’s initialization at the beginning. Training the model on a new dataset from scratch in this way often takes a long time to make the function converge. By using transfer learning techniques, the model can perform better under the same conditions, and reducing the cost of resource consumption^45^.

In this paper, the feature extraction network trained on the ImageNet dataset is transferred to the MST-DeepLabv3+ model using transfer learning, which can enhance the model's ability to obtain features and effectively improve the model segmentation accuracy.

### Accuracy evaluation

In this paper, we use the visual comparison of segmentation results and common evaluation metrics to comprehensively evaluate the model segmentation performance. The evaluation metrics used are MIoU, OA, Precision, Recall, and F1-Score.

MIoU is the most commonly used metric in semantic segmentation experiments. Its value is calculated by first calculating the ratio between the intersection and the concatenation of the two sets of true and predicted values on each category, and then finding the average of all categories. As shown in Eq. (4).

4 $$ MIoU = \frac{1}{{{\text{k}} + 1}}\sum\nolimits_{i = 0}^{k} {\frac{TP}{{FN + FP + TP}}} , $$

OA is the proportion of properly identified pixels to all pixels, which can represent the overall accuracy of the model. As shown in Eq. (5).

5 $$ OA = \frac{TP + TN}{{TP + TN + FP + FN}}, $$

Precision indicates the number of true positive pixels in the pixels that are predicted to be positive. As shown in Eq. (6).

6 $$ P{\text{recision}} = \frac{TP}{{TP + FP}}, $$

Recall is the ratio of the model’s correctly predicted positive pixels to the total positive pixels. As shown in Eq. (7).

7 $$ {\text{Re}} call = \frac{TP}{{TP + FN}}, $$

F1-Score is the harmonic mean of Precision and Recall, which is a comprehensive evaluation metric. It can solve the problem that when the number of pixels in each category deviates greatly, the OA index cannot accurately evaluate the specific classification results**.** Its equation is as follows:

8 $$ F = \frac{{2{\text{Precison}} \times {\text{Re}} call}}{{\Pr ecison + {\text{Re}} call{\text{l}}}} = \frac{2TP}{{2TP + FN + FP}}, $$

In the above equations, *k* + *1* represents the number of data categories, including the background categories. *TP* is True Positive (The model predicts a positive case, and the actual case is positive), *FP* is False Positive (The model predicts a positive case, but the actual case is negative), *FN* is False Negative (The model predicts a negative case, but the actual case is positive), *TN* is True Negative (The model predicts a negative case, and the actual case is negative).

## Experimental results and analysis

The operating system is CentOS7.9, the CPU is AMD EPYC 7402 48@ 2.8GHz, the GPU is 8*NVDIA®GeForce®RTX 3090, and the video memory is 8*24GB. The deep learning framework used is pytorch3.6. The batch_size is set to 8 and the number of iterations is set to 100. Experiments have proven that when the number of iterations reaches the maximum, the loss function has converged and the accuracy is no longer significantly improved. The basic learning rate is set to 0.0005, and the Adam optimizer is used to dynamically adjust the learning rate to make the learning rate closer to the parameter update state, thereby allowing the model to converge better.

To validate MST-DeepLabv3+’s effectiveness, it was compared to DeepLabv3+, PSPNet, and UNet, in terms of accuracy and segmentation details. UNet can achieve higher segmentation accuracy while using less data^25^. The pyramid pooling module, used by PSPNet, may aggregate contextual information from different regions, making it easier to gather global information^28^.

### Experimental results of ISPRS dataset

Table 2 statistically compares the evaluation results of MST-DeepLabv3+ on the ISPRS dataset to those of the other three models. In the comparison of the results of MIoU, OA, Precision, Recall, and F1-score, MST-DeepLabv3+ obtained the highest values. In the MIoU comparison, MST-DeepLabv3+ has a MIoU of 82.47%, which is 14.13%, 10.48%, and 13.85% higher than PSPNet, UNet, and DeepLabv3+, respectively. In the OA comparison, MST-DeepLabv3+ has an OA value of 92.13%, which is 5.38%, 4.98%, and 6.02% higher than other three models, respectively.

**Table 2:** Segmentation results on the ISPRS dataset.

Method	MIoU (%)	OA (%)	Precision (%)	Recall (%)	F1-score(%)
PSPNet	68.34	86.75	80.67	80.98	80.82
UNet	71.99	87.15	84.53	81.91	83.20
DeepLabv3+	68.62	86.11	81.80	80.13	80.96
MST-DeepLabv3+	82.47	92.13	90.34	90.12	90.23

Precision and Recall measure the correctness and completeness of segmentation, respectively, and the ideal segmentation situation is one in which both Precision and Recall are high. The Precision value of MST-DeepLabv3+ was 90.34%, the recall rate was 90.12%, and the F1-score reached the highest value of 90.23%.

Table 3 shows the specific classification results of the ISPRS dataset to further demonstrate the effectiveness of MST-DeepLabv3+. In the MIoU comparison, MST-DeepLabv3+ has the highest MIoU of all types. For the background, car, and low vegetation categories, the MIoU values of the PSPNet, UNet, and DeepLabv3+ are relatively low, MST-DeepLabv3+’s MIoU values for these three classes are 84.04%, 70.02%, and 77.51%, which are 26.14%, 24.82%, and 12.19% higher than PSPNet, 11.47%, 19.14%, and 12.84% higher than UNet, 27.89%, 17.07%, and 14.01% higher than DeepLabv3+. MST-DeepLabv3+ also has the highest MIoU values for the impervious surface, tree, and building of all types of methods.

**Table 3:** Comparison of IoU(%) for the ISPRS dataset.

Method	Background	Impervious surface	Tree	Building	Car	Low vegetation
PSPNet	57.90	79.54	74.98	87.21	45.20	65.32
UNet	72.57	81.02	74.89	87.91	50.88	64.67
DeepLabv3+	56.15	78.82	73.91	86.37	52.95	63.5
MST-DeepLabv3+	84.04	87.80	82.17	93.28	70.02	77.51

**Figure 6:**
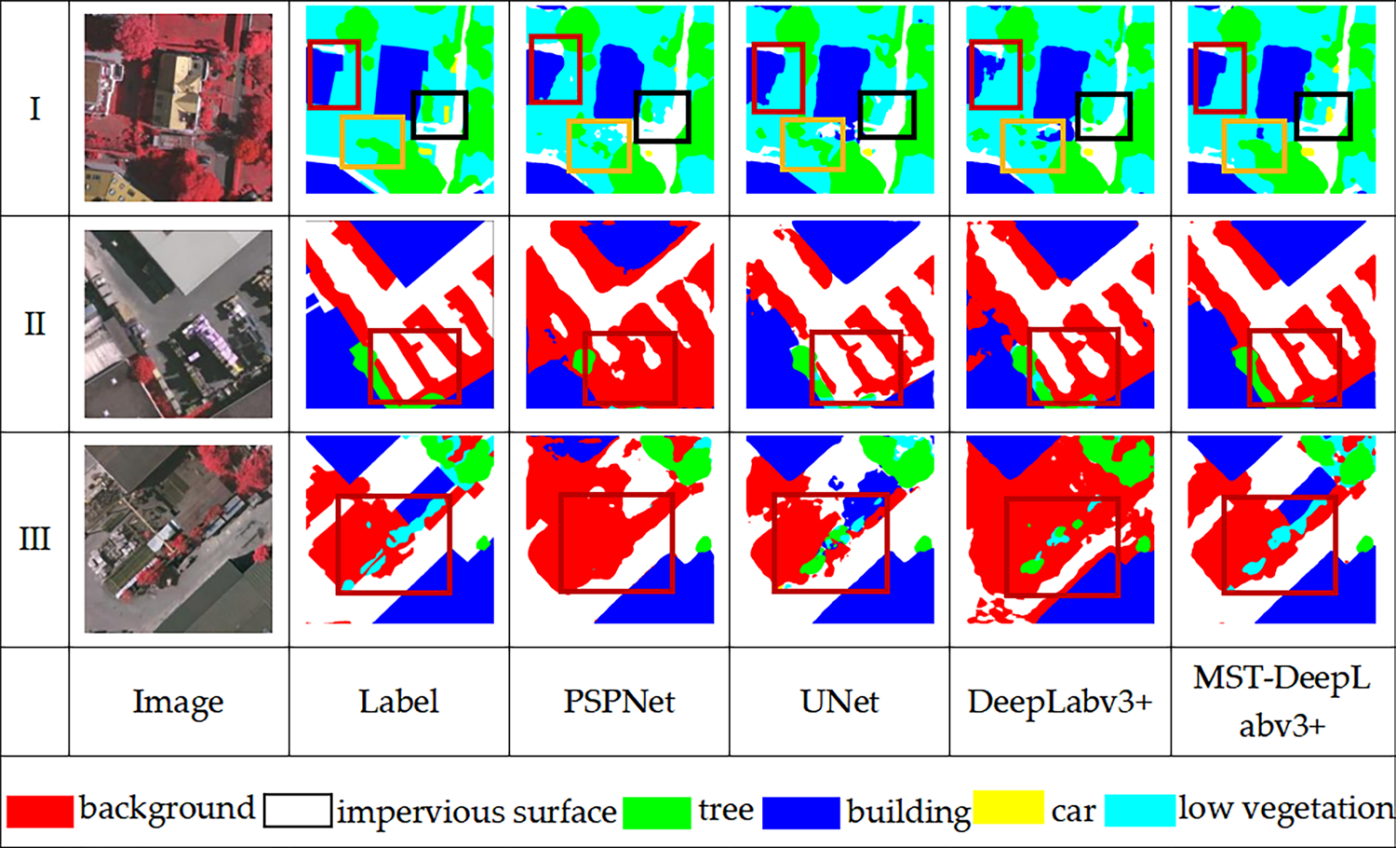
Example of classification result visualization of ISPRS dataset.

Three cropped images were analyzed to further compare the classification results of different models. As shown in Fig. 6.

In group I, The models PSPNet, UNet, and DeepLabv3+ all missed the small car patches in the black box, only the MST-DeepLabv3+ can correctly identify the overall contour and location. For the tree shown in the yellow box, compared with the classical model segmentation result of scattered and no general outline, MST-DeepLabv3+ model can accurately identify the category regions with complete boundaries. The building segmentation results in the red box show that the classical model is not accurate for contour identification, especially the DeepLabv3+ model segmentation results, which are fragmented and the boundary is obviously incorrect. However, MST-DeepLabv3+, on the other hand, accurately identifies the boundary between buildings and low vegetation.

In group II, the building, impervious surface and background areas are regularly arranged, and the boundary contrast is more obvious. PSPNet and DeepLabv3+ cannot identify the impervious surface and the background boundary, and the impervious surface and the building boundary better. PSPNet is worse and identifies most of the impervious surface areas as background and building. The segmentation boundaries of UNet and DeepLabv3+ are rough. MST-DeepLabv3+ not only has the best segmentation effect but also has smoother edges.

In group III, the region shown in the red box is the segmentation result comparison of the low vegetation, PSPNet does not identify the low vegetation at all, the segmentation results of UNet and DeepLabv3+ are fragmented, and the low vegetation were misclassified into the tree. In addition, the results of the DeepLabv3+ also have a large area of impervious surface misclassified into background. MST-DeepLabv3+ can completely identify the overall region of the category, and the segmentation effect is the best.

Overall, in terms of classification effect, MST-DeepLabv3+ outperforms PSPNet, UNet, and DeepLabv3+.

### Experimental results of GID dataset

Table 4 compares the classification accuracy evaluation results of the four models on the GID dataset. In the comparison results of MIoU, Recall, OA, and F1-score, MST-DeepLabv3+ all obtained the highest values. In the MIoU comparison, the MIoU of MST-DeepLabv3+ is 73.44%, which is 1.56%, 2.2%, and 7.11% higher than PSPNet, UNet, and DeepLabv3+, respectively. In the OA comparison, MST-DeepLabv3+ has the highest OA value of all models at 85.58%.

**Table 4:** Segmentation results on the GID dataset.

Method	MIoU (%)	OA (%)	Precision (%)	Recall (%)	F1-score (%)
PSPNet	71.88	84.91	85.39	81.66	83.48
UNet	71.24	83.50	83.92	81.99	82.94
DeepLabv3+	66.33	80.51	78.81	80.11	79.45
MST-DeepLabv3+	73.44	85.58	84.10	84.86	84.48

MST-DeepLabv3+ has a Precision value of 84.10%, which is higher than UNet and DeepLabv3+ but slightly lower than PSPNet. MST-DeepLabv3+ has the highest Recall of 84.86%, with an F1-score of 84.48%. The F1-score is 1%, 1.54%, and 5.03% higher than the PSPNet, UNet, and DeepLabv3+ models, respectively.

Table 5 displays the specific classification results of the GID dataset. In the MIoU comparison, MST-DeepLabv3+ has the highest MIoU in five types: background, water, farmland, build-up, and forest. And DeepLabv3+ has the lowest IoU of the five types. UNet has the highest accuracy in the meadow category, which is 1.75% higher than MST-DeepLabv3+.

**Table 5:** Comparison of IoU(%) for the GID dataset.

Method	Background	Water	Farmland	Built-up	Meadow	Forest
PSPNet	71.33	88.44	76.05	69.62	63.85	61.97
UNet	69.03	88.73	72.99	67.05	67.98	61.64
DeepLabv3+	63.76	84.71	70.20	64.59	62.11	52.6
MST-DeepLabv3+	71.91	88.86	77.32	70.09	66.23	66.24

**Figure 7:**
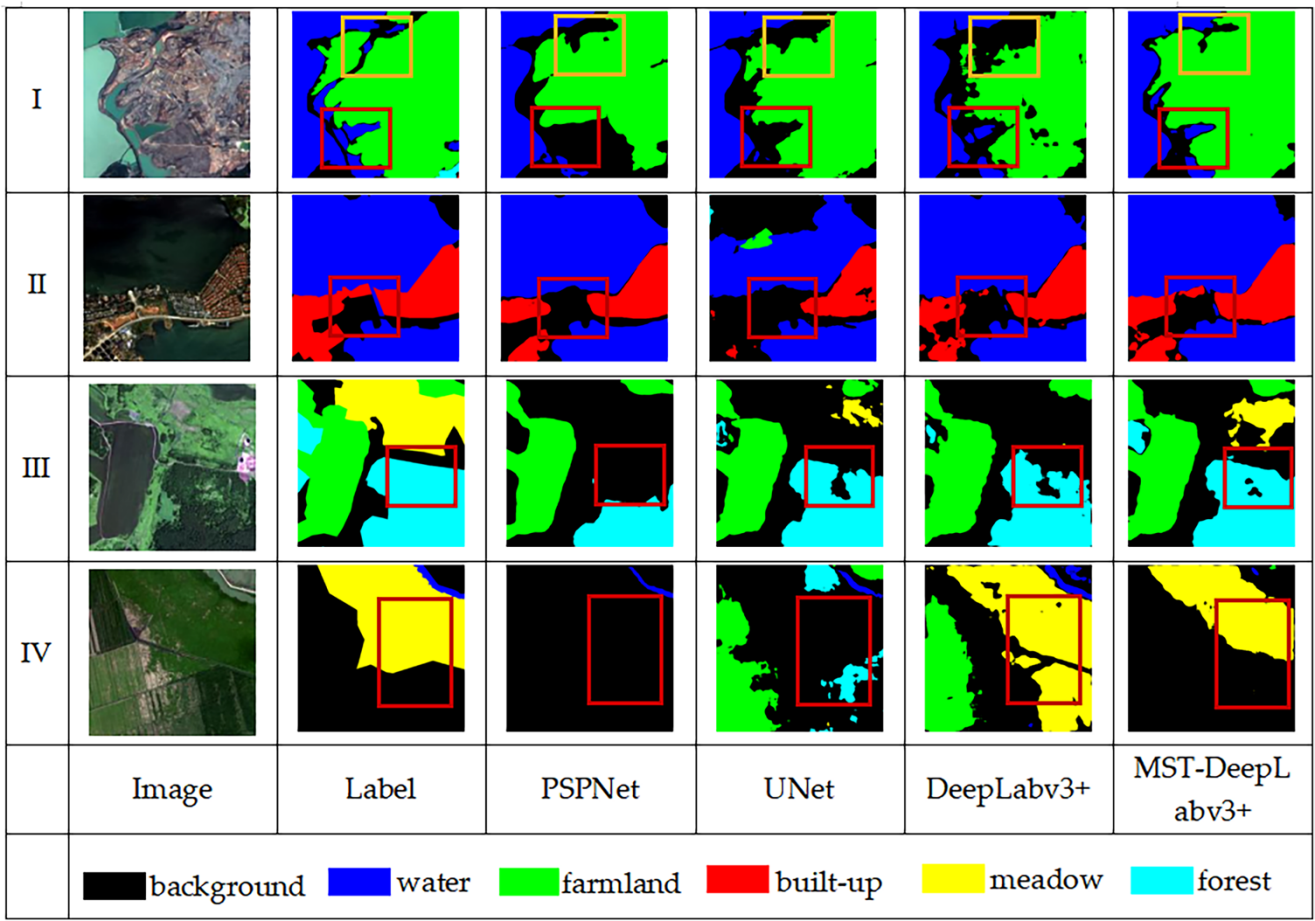
Example of classification result visualization of GID dataset.

Four cropped images were selected to show the visualization of semantic segmentation results of different models on the GID dataset. As shown in Fig. 7.

In group I,it is mainly the boundary division of the farmland that occupies most of the area from the water and the background. As shown in the yellow box, PSPNet, UNet, and DeepLabv3+ models have poor recognition of farmland and background, in which DeepLabv3+ is especially obvious and the overall region segmentation is more fragmented. MST-DeepLabv3+ is relatively accurate for the boundary recognition of farmland and background. The region shown in the red box is the water category segmentation result, which clearly shows that PSPNet and UNet incorrectly identify water as background, while DeepLabv3+ recognizes part of the water region, but the boundary is incomplete, MST-DeepLabv3+ identifies the boundary between water and background more accurately, as well as the boundary between water and farmland.

In group II, PSPNet, UNet and DeepLabv3+ can hardly recognize the small patch area of built-up, but MST-DeepLabv3+ can identify it accurately and optimizes the category segmentation range.

In group III, PSPNet recognizes forest as background, UNet and DeepLabv3+ can recognize a portion of the forest's outline, but the edges are rough. MST-DeepLabv3+ identifies the entire forest region more effectively, and the edges are smoother and more continuous.

In group IV, PSPNet completely fails to recognize the meadow, UNet incorrectly recognizes meadow as forest, and DeepLabv3+, which is oversegmented. In comparison, MST-DeepLabv3+ extracts more information on the meadow.

In general, MST-DeepLabv3+ outperforms other models in terms of classification effect, improves the phenomenon of incomplete classification, unclear boundary, misclassification, omission, and over-segmentation, and significantly improves recognition accuracy.

### Experimental results of Taikang cultivated land dataset

Table 6 shows the segmentation results of each model on the Taikang cultivated land dataset. Among the classic models, UNet has the highest values of all evaluation metrics. The MIoU of MST-Deeplapv3+ reaches 90.77%, which is 3.71% higher than UNet. The OA, Precision, Recall and F1-score of MST-Deeplapv3+ reach 95.47%, 95.28%, 95.02%, and 95.15%, which are 1.94%, 2.06%, 2.21%, and 2.09% higher than UNet, respectively. PSPNet has the lowest values for all metrics, with MIoU lower than MST-DeepLabv3+ by 5.94%, and OA, Precision, Recall and F1-score lower than MST-Deeplapv3+ by 3.17%, 3.44%, 3.36%, and 3.40%, respectively. Compared to DeepLabv3+, the specific improvement of each evaluation value of MST-DeepLabv3+ is 5.37% for MIoU, 2.83% for OA, 2.91% for Precision, 3.19% for Recall, and 3.05% for F1-score.

**Table 6:** Segmentation results on the Taikang cultivated land dataset.

Method	MIoU (%)	OA (%)	Precision (%)	Recall (%)	F1-score (%)
PSPNet	84.83	92.30	91.84	91.66	91.75
UNet	87.06	93.53	93.22	92.9	93.06
DeepLabv3+	85.40	92.64	92.37	91.83	92.10
MST-DeepLabv3+	90.77	95.47	95.28	95.02	95.15

The comparison of segmentation results of Taikang cultivated land dataset is shown in Table 7. The comparison results show that MST-DeepLabv3+ has the best segmentation effect, and the IoU of cultivated land reaches 93.06%, which is an increase of 4.59%, 2.83% and 4.05% compared to PSPNet, UNet and DeepLabv3+ models, respectively. The IoU for background categories reached 88.48%, which is an increase of 7.28%, 4.59% and 6.68% compared to PSPNet, UNet and DeepLabv3+, respectively.

**Table 7:** Comparison of IoU(%) for the Taikang cultivated land dataset.

Method	Background	Cultivated land
PSPNet	81.20	88.47
UNet	83.89	90.23
DeepLabv3+	81.80	89.01
MST-DeepLabv3+	88.48	93.06

**Figure 8:**
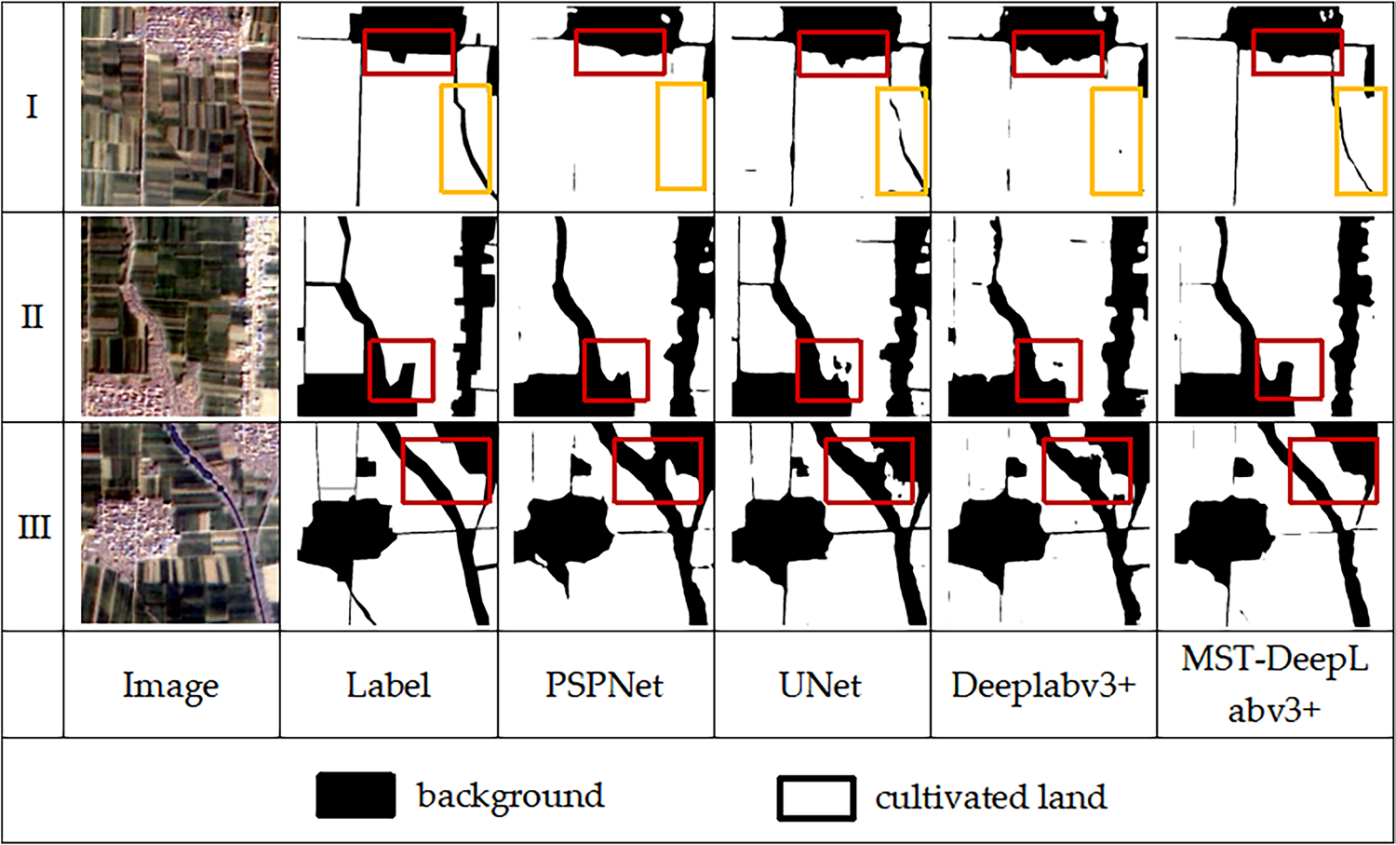
Example of classification result visualization of Taikang cultivated land dataset.

Three groups of images of cultivated land segmentation results are selected for comparison and analyzed with the specific information of background categories in the images. As shown in Fig. 8.

In group I, the red box shows the segmentation of the residential area bordering with the cultivated land. PSPNet can hardly recognize the small patch residential area.UNet and DeepLabv3+ can roughly recognize this area, but there is a rough and unsmooth border segmentation of the cultivated land.MST-DeepLabv3+ recognizes the cultivated land and the background in this area more clearly. The yellow box, is the distinction between the boundary of the cultivated land and the road. PSPNet and DeepLabv3+ can’t recognize the boundary. The segmentation result of UNet is not continuous, and the middle of the road produces a discontinuity.MST-DeepLabv3+ can recognize it accurately.

In group II, the background category is unused land in non-residential areas. PSPNet, UNet and DeepLabv3+ all have misclassification phenomena at the intersection between the background and cultivated land, and the contours are inaccurate. The segmentation result of MST-DeepLabv3+ is closer to the label.

In group III, for the segmentation between the cultivated land and the road on both sides of the river, PSPNet, UNet and DeepLabv3+ all showed the phenomenon of adhesion. MST-DeepLabv3+ can effectively improve this phenomenon with clear boundaries.

In conclusion, MST-DeepLabv3+ is able to effectively optimize the phenomena of rough boundaries, inaccurate contour prediction, and adhesion between categories that occur in other model segmentations.

### Ablation experiment and model parameter comparison

The ablation experiment can demonstrate the changes of the model itself and the segmentation effect during each step of the improvement process. In this paper, the most commonly used MIoU metrics and model parameter size metrics are selected to illustrate the model improvement process. Since the training time is closely related to the parameter size, it is also affected by subjective factors such as the experimental platform and training environment. Therefore, this article only describes the size of the model parameters, not the training time. The results of ablation experiments based on the ISPRS dataset are shown in Table 8. The parameter size of DeepLabv3+ model with Xception as the backbone network is 208.7 MB. After replacing the Xception network with MobileNetV2 network, the model parameters are reduced to 22.19 MB, and the MIoU value is also reduced by 4.36% due to the impact of the lightweight network. After adding the attention mechanism SENet, the model parameters increased slightly by 0.77 MB, but the MIoU increased by 5.35%, which nicely fills the accuracy loss in the previous step. The addition of transfer learning does not change the size of the model parameters, which again significantly improves the segmentation accuracy of the model.

**Table 8:** Ablation experiment.

DeepLabv3+	Backbone		SENet	Transfer learning	MIoU (%)	Parameter amount (MB)
	Xception	MobileNetV2				
√	√				68.62	208.7
√		√			64.26	22.19
√		√	√		69.61	22.96
√		√	√	√	82.47	22.96

The size of model parameters is the main factor of image training efficiency. The smaller the number of model parameters, the shorter the training time, which can effectively improve the model training speed. Table 8 compares the parameter size changes during model improvement through ablation experiments, and Table 9 shows the parameter comparison of the MST-DeepLabv3+ model with other models. The parameter size of the MST-DeepLabv3+ model is 22.96 MB, which is about 91% reduction compared to the PSPNet model and about 76% reduction compared to the UNet model. The parameters of MST-DeepLabv3+ model are much lower than the PSPNet, UNet and DeepLabv3+ models.

**Table 9:** Comparison of parameter sizes for different models.

Method	Parameter amount (MB)
PSPNet	259.64
UNet	94.95
DeepLabv3+	208.70
MST-DeepLabv3+	22.96

## Discussion

Due to the vast scene, complicated details, and effects of illumination and imaging angle in high-resolution remote sensing images, classic semantic segmentation models frequently have issues, such as low training efficiency, inaccurate target recognition, and low accuracy. We propose the MST-DeepLabv3+ semantic segmentation model to solve these problems. This model fully integrates the advantages of lightweight network, attention mechanism, and transfer learning to provide the best performance in processing remote sensing images.

Firstly, the lightweight network is applied in the model to reduce the number of model parameters. Assuncao et al.^46^ used MobileNet as the DeepLabv3 model's backbone network for semantic segmentation of crops and weeds, which effectively increased the speed of model execution segmentation. Huang et al.^47^ used MobileNetV1 and MobileNetV2 instead of various models' backbone networks to reduce network training time. When the input dimension is low, the ReLU activation function used by MobileNetV1 loses more information^36^, whereas MobileNetV2 uses Linear bottleneck and Inverted residuals to maximize information retention. MST-DeepLabv3+ uses the lightweight network MobileNetV2 to replace the Xception network used for feature extraction, which greatly reduces model parameter and memory consumption, and improves model training speed. That is supported by the experimental results. When using MobileNetV2 as the backbone network, the model’s parameter size is only 22.19 MB, about one-tenth the size of DeepLabv3+, effectively reducing training consumption.

Secondly, SENet is introduced to the encoding part to distribute channel weight, so that the network starts from global information and makes up for the accuracy loss caused by the lightweight feature extraction network. At present, there are other types of attention mechanisms applied to remote sensing image segmentation. Liu et al.^48^ embedded DAMM (Dual Attention Mechanism Module) into the model to improve urban building detection in remote sensing images. Wang et al.^49^ introduced CBAM (Convolutional Block Attention Module) into the model to improve road detection performance in high-resolution remote sensing images. The DAMM contains a position attention module that mainly considers the global information of fusion features, which is similar to the function of DeepLabv3+’s ASPP module. Although CBAM has both spatial and channel attention modules, it cannot make reasonable use of spatial information at different scales. We added the SENet module to make the model portable and effective, and the model parameter size increased from 22.19 to 22.96 MB. When classifying the ISPRS dataset, MIoU is increased from 64.26% to 69.61%, the accuracy is significantly improved with a small increase in computational effort.

Finally, transfer learning is introduced into the model feature extraction network, and the pre-training model parameters are used as the initial weight parameters of the network, which can make the model segmentation effect better.

Combined with the preceding three points, MST-DeepLabv3+ achieves MIoU of 82.47%, 73.44%, and 90.77% on the ISPRS dataset with aspatial resolution of 9 cm, the GID dataset with a aspatial resolution of 1m, and the Taikang cultivated land dataset with a aspatial resolution of 2 m, respectively. The segmentation accuracy is improved, the whole and detailed information of the high-resolution remote sensing image is better identified, and the final model parameter size is 22.96 MB, significantly improving training efficiency.

## Conclusions

This paper proposes a remote sensing image classification algorithm to address the problems of low precision and low model training efficiency in remote sensing image semantic segmentation. Replace the DeepLabv3+ model’s backbone network with MobileNetV2 to decrease the number of model parameters and memory occupation to speed up training; add an attention mechanism to make up for the accuracy loss brought on by the lightweight feature extraction network and improve the model's deficiency in capturing ground information; introduce the transfer learning method and use the pre-training model parameters as the network's initial weight parameters to improve the model segmentation effect. The classification results of the ISPRS dataset, GID dataset, and Taikang cultivated land dataset show that MST-DeepLabv3+ can effectively improve segmentation accuracy and training efficiency, and its overall performance is the best among the compared models.

Aiming at the problem of insufficient boundary information extraction that still exists in the experiment, the next work can combine the edge extraction model with the semantic segmentation model to optimize the segmentation boundary. Simultaneously, the generalization and learning migration capability of the model needs to be improved for remote sensing image segmentation with different terrains. In addition, MST-DeepLabv3+ does not consider multispectral information, and adding spectral information may improve segmentation precision.

## Data Availability

The processed ISPRS dataset in the current study can be downloaded from the following link: https://www.scidb.cn/en/s/ERFnAb. The processed GID dataset in the current study can be downloaded from the following link: https://www.scidb.cn/en/s/eaiY7f. The Taikang cultivated land dataset used in the current study is not publicly available due to its current confidential status, but is available from the corresponding author on reasonable request.
